# Supplementary material for: Electron shelving of a superconducting artificial atom
Source: Nat Commun. 2021 Nov 4;12:6383. doi: 10.1038/s41467-021-26686-x (PMC8569191; doi:10.1038/s41467-021-26686-x)
Supplement: Supplementary file 1 — Supplementary Information [file 41467_2021_26686_MOESM1_ESM.pdf]

# Supplementary Information for
# "Electron shelving of a superconducting artificial atom"

Nathanaël Cottet[1,2], Haonan Xiong[2], Long B. Nguyen[2], Yen-Hsiang Lin[2], and Vladimir E. Manucharyan[2]

[1]Physics Department, University of Maryland, College Park, MD 20742
[1]Université Lyon, ENS de Lyon, Université Claude Bernard Lyon 1, CNRS, Laboratoire de Physique, F-69342 Lyon, France
[2]Physics Department, University of Maryland, College Park, MD 20742

## Supplementary Note 1: RF-reflectometry setup

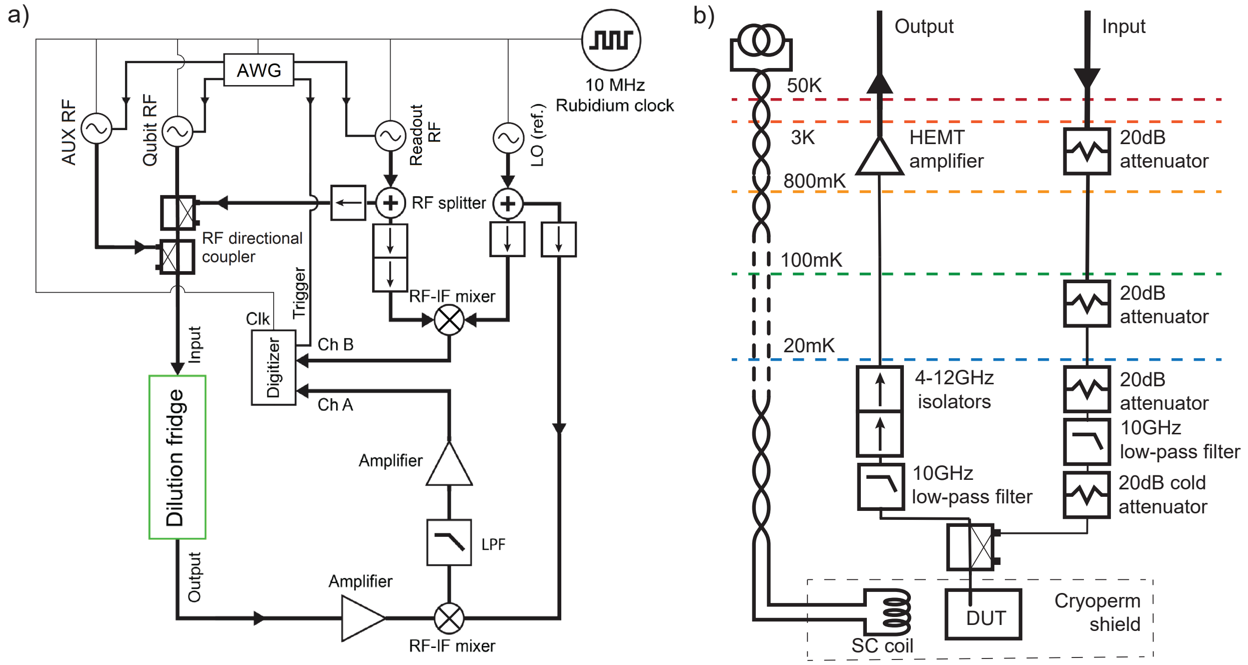


**Supplementary Figure** 1: **Experimental setup** (a) Room-temperature signal generation and acquisition. (b) Cryogenic experimental setup inside the dilution refrigerator.

Schematic of our radio-frequency (RF) measurement setup is shown in Supplementary Fig. 1. At room temperature, we utilized three Rohde & Schwarz RF-sources to generate the readout tone (denoted by Readout RF), the qubit $|0\rangle$-$|1\rangle$ tone (Qubit RF), and the auxiliary $|0\rangle$-$|2\rangle$ tone (AUX RF). The CW-signals are directly modulated by the analog outputs of a Tektronix arbitrary waveform generator (AWG) with sampling rate of 1 GSa/s, which also triggers the Alazar signal digitizer. The modulated signals from all RF sources are combined using two directional couplers. To maximize the available power of the qubit tone, we connected Qubit RF to the input ports of the directional couplers while the pumping tone and readout tone go to the couple ports with 20 dB isolation. The combined signal is directed into the dilution refrigerator as the input signal. The signal coming from the refrigerator gets amplified first and then is down-converted by mixing it with a reference tone (LO) detuned from Readout RF by 50 MHz, filtered and amplified before digitization (ch A). Its phase is compared to the phase of a reference (ch B), obtained by down-converting a part of the readout RF that does not go through the fridge. The isolators after splitters are used to suppress the leakage from the mixers and the reflection from the directional coupler because these leakage can bring systematic error to the measurement of the reflection coefficient.

The cryogenic wiring is shown in Supplementary Fig. 1b. The input coaxial line contains several cryogenic 20 dB attenuators at the 4 K, 100 mK and 10 mK stages of the dilution refrigerator. In addition, we used a K&L low-pass filter at 10mK with a cut-off frequency of 10 GHz, followed by a home-made 20 dB cold attenuator, the details of which will be described in a separate publication. The input line connects to the sample holder port via a directional coupler. The reflected signal goes out through a chain containing K&L

filter (cut-off frequency of 10 GHz), two 4-12 GHz isolators, and a low-temperature HEMT amplifier. The 10 mK space was protected using a radiation shield and the device was inside a single layer of a cylindrical Cryoperm magnetic shield.

## Supplementary Note 2: Spectroscopy and circuit parameters

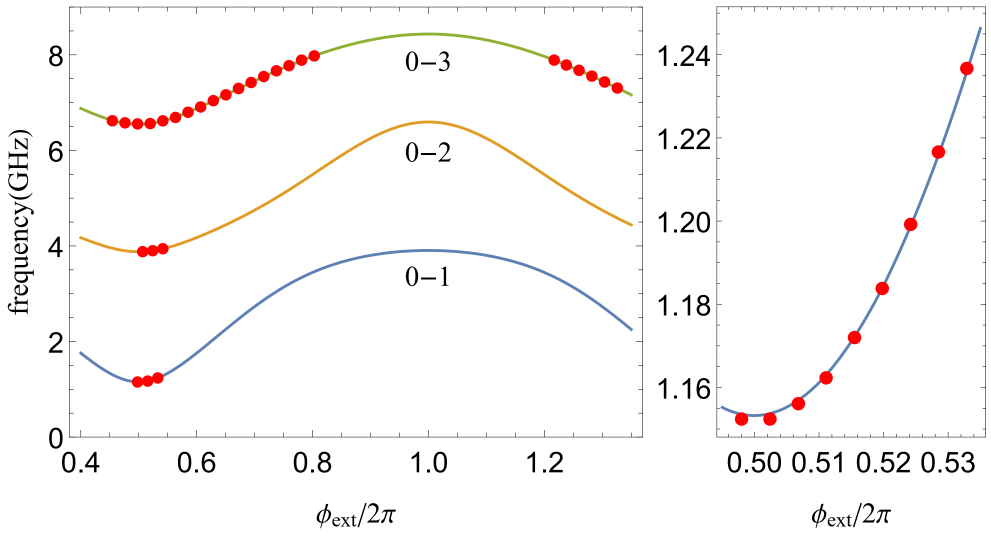


**Supplementary Figure** 2: **Fluxonium spectroscopy.** Red dots are experimentally measured transitions and plain lines are the fit from diagonalization of $H_f$. The right figure shows $|0\rangle$-$|1\rangle$ around the sweet spot.

Fluxonium circuit is represented by the Hamiltonian [5]

$$H_f = 4E_C(-i\partial\phi)^2 + E_L\phi^2 - E_J\cos(\phi - \phi_{\rm ext}) \tag{1}$$

with $E_J$ the Josephson energy of the weak junction, $E_C = \frac{e^2}{2C}$ the charging energy coming from the shunting capacitance $C$ and $E_L$ the inductance energy of the chain. The measured fluxonium spectrum is shown in Supplementary Fig. 2 (red dots) along with with the fit (plain lines), yielding $E_C = 2\pi \times 1.2$ GHz, $E_L = 2\pi \times 0.62$ GHz and $E_J = 2\pi \times 2.0$ GHz. These parameters were used to numerically calculate the matrix elements of charge and phase operators.

## Supplementary Note 3: Qubit gate fidelity

We illustrate the high degree of control over the qubit state using randomized benchmarking (see Supplementary Fig. 3). The Clifford gates are realized using a succession of gaussian pulses of width 187 ns and total duration 374 ns, performing $\pi$ or $\pi/2$ rotations of the qubit. For the sequence used here, a Clifford gate contains on average 1.875 pulses. The data were fitted with the zeroth-order model [7] $p_0 = Ap^m + B$, where $m$ is the number of Clifford gates and $p$ is the depolarizing parameter. For a single qubit, the average Clifford gate error is given by $r = (1-p)/2$. The fit of the data yields to the average pulse fidelity $F_{01} = 1 - r/1.875 = 0.9942 \pm 0.0002$. We note that the relatively slow gate operation was due to a suboptimal

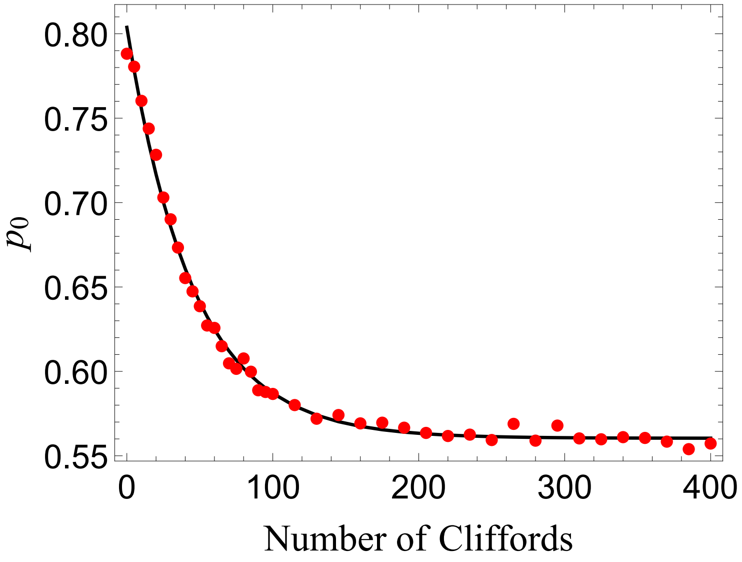


**Supplementary Figure** 3: **Randomized benchmarking on single qubit gates for $|0\rangle$-$|1\rangle$.** Red dots are experimentally measured population corresponding to different number of Clifford gates and plain lines are the fit from the zeroth-order model.

positioning of the chip with respect to the coaxial-to-waveguide adaptor, which resulted in an excessive undercoupling at the qubit frequency. In a future work, the gate speed can be increased by orders of magnitude without affecting the qubit coherence time by moving the chip closer to the launcher.

## Supplementary Note 4: Readout histograms

In order to estimate the possibility of single-shot readouts based on conditional fluorescence, we measured the fluorescence histograms at thermal equilibrium and after a $\pi$-pulse between $|0\rangle$ and $|1\rangle$. They are represented on Supplementary Fig. 4, for $10^5$ realizations. We fit each histogram by a Gaussian distribution function $g(x) = \exp\left(-(x - x_0)^2/(2\sigma^2)\right)$ and find a relative separation

$$x_\pi - x_{\text{th}} = 0.33 \times \frac{s_\pi + s_{\text{th}}}{2} \tag{2}$$

with $x_{\text{th}}, s_{\text{th}}$ (resp. $x_\pi, s_\pi$) the mean and standard deviation at thermal equilibrium (resp. after a $\pi$-pulse). Such a separation can be increased by a factor 10 by adding a quantum-limited amplifier after the waveguide [6, 1], leading to well separated histograms, thus single-shot readouts.

## Supplementary Note 5: Time-domain control of $|0\rangle$-$|2\rangle$ transition

By operating at a flux slightly away from the sweet spot we were able to break the parity selection rule, which allowed us to directly drive even transitions. Applying a gaussian pulse at $\omega_{02} = 2\pi \times 3.88$ GHz of varying width results in Rabi oscillations of the $|0\rangle$-$|2\rangle$ transition, represented on Supplementary Fig. 5a. A swap between $|0\rangle$ and $|2\rangle$ is realized for a pulse of width 196 ns and total duration of $t^{02}_{\text{total}} = 392$ ns. In order to estimate the fidelity of the swap, we measured the coherence time of this transition by Ramsey interferometry

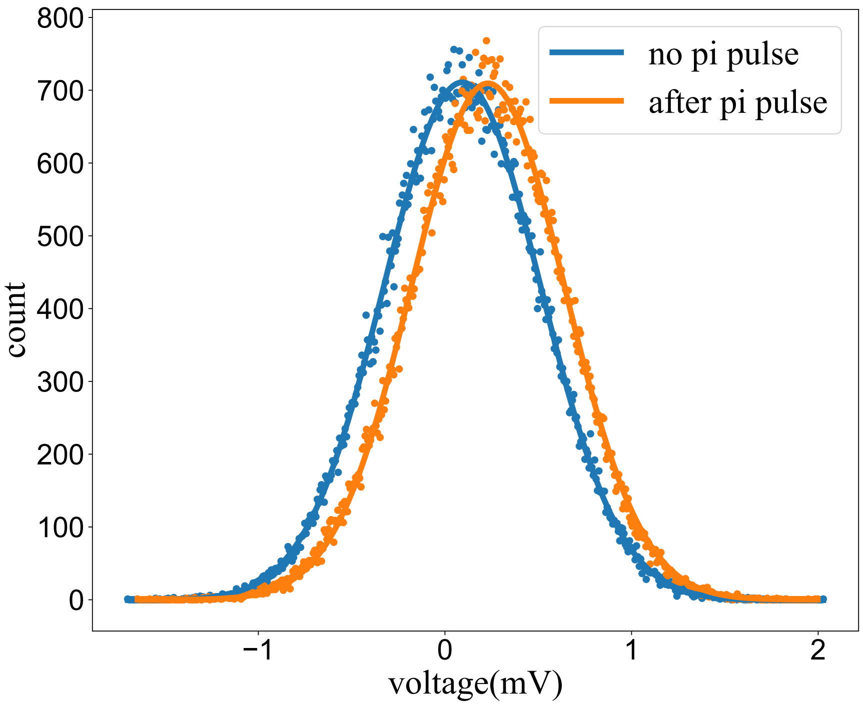


**Supplementary Figure 4: Histograms of conditional fluorescence.** Measured (dots) and fitted (plain lines) histograms for $10^5$ realizations of the readout for the circuit at thermal equlibrium (blue) or after a swap between $|\,|0\rangle$ and $|1\rangle$ (orange).

(Supplementary Fig. 5b), leading to $T_2^{02} = 1.6\ \mu$s. The lifetime $T_1^{02}$ is estimated from the computed decay rates in Supplementary Table. 1. At first order, the pulse fidelity can be estimated from the loss of contrast of Rabi oscillations. For $t_{\rm total}^{02} \ll T_1^{02}, T_2^{02}$ we obtain the estimated fidelity $F_{02} \approx 1 - t_{\rm total}^{02}(1/T_1^{02} + 1/T_2^{02})/4 = 0.93$.

## Supplementary Note 6: Qubit initialization by optical pumping of $|1\rangle$-$|3\rangle$ transition

With the ability to drive the even transitions at this flux point, we could prepare the ground state by driving the transition $|1\rangle$-$|3\rangle$ as presented in Supplementary Fig. 6. By design of the readout transition, $|3\rangle$ decays to $|0\rangle$ much faster than to other states. Therefore if thermal population gets pumped to $|3\rangle$, most of it decays to $|0\rangle$. In this way, we could prepare $p_0$ to be 0.9 after 5 $\mu$s pumping, limited by how quickly we can drive the pumping transition $|1\rangle \rightarrow |3\rangle$. In theory, the maximal value of $p_0$ approaches unity upon increasing $T_1$ and reducing the error rate of decay from state $|3\rangle$ to state $|1\rangle$.

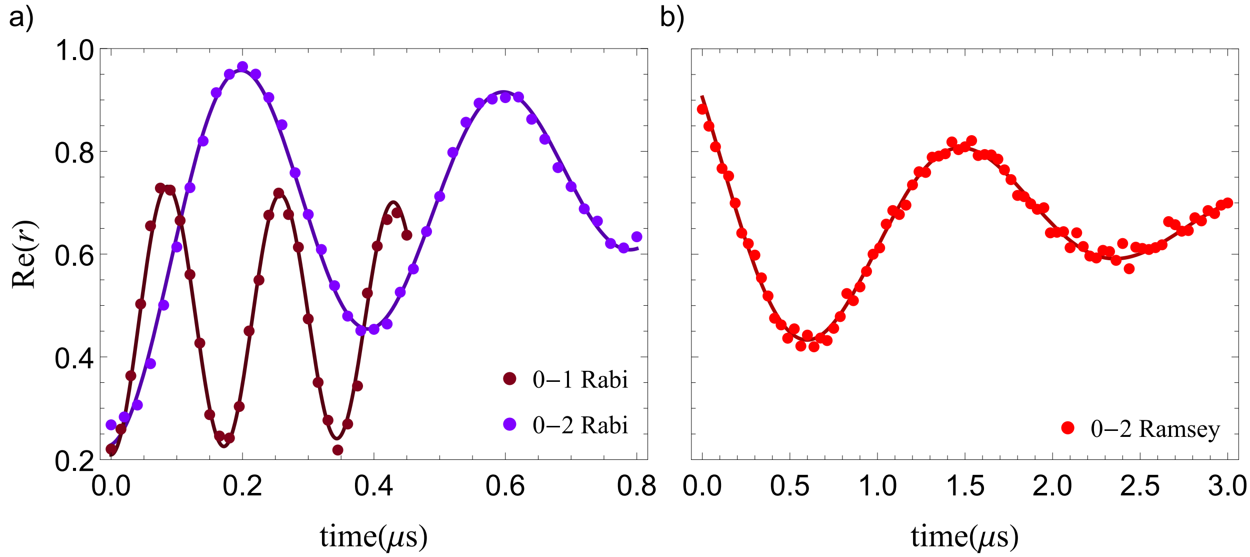


**Supplementary Figure** 5: **Coherent manipulation of** $|0\rangle$**-**$|2\rangle$**.** (a) Rabi oscillations of the $|0\rangle$-$|1\rangle$ transition (brown) and $|0\rangle$-$|2\rangle$ transition (purple). (b) Ramsey oscillations obtained by directly driving the $|0\rangle$-$|2\rangle$ transition.

## Supplementary Note 7: Independent estimate of thermal population

We detail in this section how Rabi oscillations of $|0\rangle$-$|1\rangle$ and $|0\rangle$-$|2\rangle$ allow for an independent measurement of the thermal occupation of the atom. As shown in Eq. (1) of the main text, the reflection coefficient $r$ is related to the ground-state population $p_0$ by $r = 1 - \alpha p_0$, where $\alpha$ is a coefficient depending on the readout drive amplitude and frequency. Denoting $r_0,\ r_1,\ r_2$ the measured reflection coefficients when the ground state population is equal to $p_0^{\mathrm{th}},\ p_1^{\mathrm{th}}$, and $p_2^{\mathrm{th}}$ respectively, we obtEain the following relations

$$p_1^{\mathrm{th}}/p_0^{\mathrm{th}} = (1 - r_1)/(1 - r_0) \tag{3}$$

$$p_2^{\mathrm{th}}/p_0^{\mathrm{th}} = (1 - r_2)/(1 - r_0)\ . \tag{4}$$

Importantly, there is no assumption to make on the value of $\alpha$ here, therefore this measurement is independent from the estimate coming from fitting the readout spectroscopic measurements (Fig. 2 of the main text). As demonstrated in the previous section, the fidelity of $|0\rangle$-$|1\rangle$ pulses exceeds 99%. The value $r_1$ is thus simply estimated by the maximum of Rabi oscillations on $|0\rangle$-$|1\rangle$ in Supplementary Fig. 4a. It yields $p_1^{\mathrm{th}}/p_0^{\mathrm{th}} = 0.329$. In order to correct for decoherence during Rabi oscillations of $|0\rangle$-$|2\rangle$, the curve presented on Supplementary Fig. 5a is fitted by the function $r(t) = -A\cos(\Omega_{02}t)e^{-t/T_{\mathrm{R}}} + Be^{-t/T_{\mathrm{out}}} + C$. Including an exponentially decreasing mean value of the oscillations takes into account the fact that population decays outside of the Rabi oscillations subspace (here, to $|1\rangle$), and gives $r_2 = A + B + C$. From the result of the fit (plain line on Supplementary Fig. 5a) we get $p_2^{\mathrm{th}}/p_0^{\mathrm{th}} = 0.007$. Assuming $p_0^{\mathrm{th}} + p_1^{\mathrm{th}} + p_2^{\mathrm{th}} = 1$, we obtain the value $p_0^{\mathrm{th}} = 0.75{\pm}0.02$.

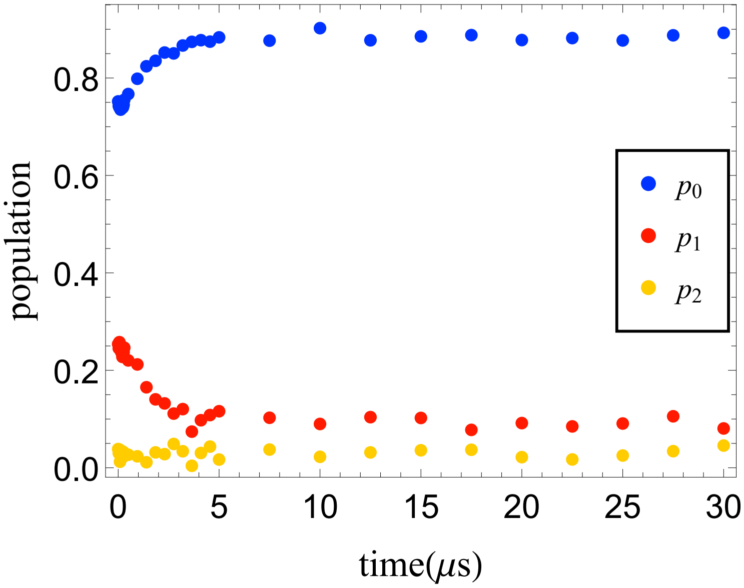


**Supplementary Figure 6**: **Qubit population initialization by pumping $|1\rangle$-$|3\rangle$.** Time-domain evolution of populations during $|1\rangle$-$|3\rangle$ pumping. 88% of the population can be prepared to $|0\rangle$ after pumping for 5 $\mu$s.

## Supplementary Note 8: Atom decay rates

The dynamics of the atom during readout or after a swap between $|0\rangle$ and $|2\rangle$ is modeled by three decay mechanisms: radiative decay in the transmission line through the waveguide, linear loss due to a lossy dielectric under the capacitance plates, and tunneling of quasiparticles in the weak junctions. Dielectric loss is the main limiting factor of transmon energy relaxation times [11]. However, energy relaxation due quasiparticle tunneling has been reported in a number of imperfectly shielded cQED systems [2, 9, 10, 8]. In principle, each mechanism could be described by a separate effective temperature, but for simplicity here we assume a common temperature of 50 mK. In particular, the temperature dependence of quasiparticle decay is captured by adding the detailed balance factor [3] in front of the expression computed in Ref. [2]. With these assumptions, the decay rates of state $|i\rangle$ to $|j\rangle$ due to dielectric loss and quasiparticle tunneling are given by

$$\Gamma_{ij}^{\mathrm{diel}} = \omega_{ij}^2 \frac{R_Q C}{Q_{\mathrm{diel}}} |\langle i|\phi|j\rangle|^2 \times \Big(1 + \coth(\frac{\hbar\omega_{ij}}{2k_B T})\Big) \tag{5}$$

$$\Gamma_{ij}^{\mathrm{qp}} = x_{\mathrm{qp}} \frac{8E_J}{\hbar\pi} \sqrt{\frac{2\Delta}{|\omega_{ij}|}} |\langle i| \sin(\frac{\phi - \phi_{\mathrm{ext}}}{2})|j\rangle|^2 \times \frac{1}{1 + e^{-\hbar\omega_{ij}/k_B T}} \tag{6}$$

with $\omega_{ij} = \omega_i - \omega_j$ the transition frequency, $C$ the shunting capacitance, $\Delta$ the superconducting gap, $R_Q$ the resistance quantum and $k_B$ the Boltzmann constant.

The waveguide coupling to the transmission line is modeled as a frequency-dependent capacitance coupling the circuit charge operator $-i\partial\phi$ to a resistor $R$ biased by a noisy voltage source $V_N$ [4]. This capacitive coupling is proportional to the power transmission coefficient $|t(\omega)|^2$ (see Fig. 1 of the main text), which is measured experimentally by adding an additional microwave port to the waveguide and measuring the

transmission. In doing so, we assume that the density of states seen by the additional port is equal to the one seen by the circuit. The resistor noise spectral density at frequency $\omega$ is $2\hbar\omega R$ [3]. Applying the Fermi Golden Rule and keeping the explicit dependence on temperature yields

$$\Gamma_{ij}^{\mathrm{rad}} \propto |\omega_{ij}||t(|\omega_{ij}|)|^2 |\langle i| - i\partial\phi|j\rangle|^2 \times \left(1 + \coth(\frac{\hbar\omega_{ij}}{2k_B T})\right) . \tag{7}$$

The scaling factor is determined experimentally from the atom spectroscopy represented on Fig. 2 of the main text and giving $\Gamma = \Gamma_{03} + \Gamma_{30}$.

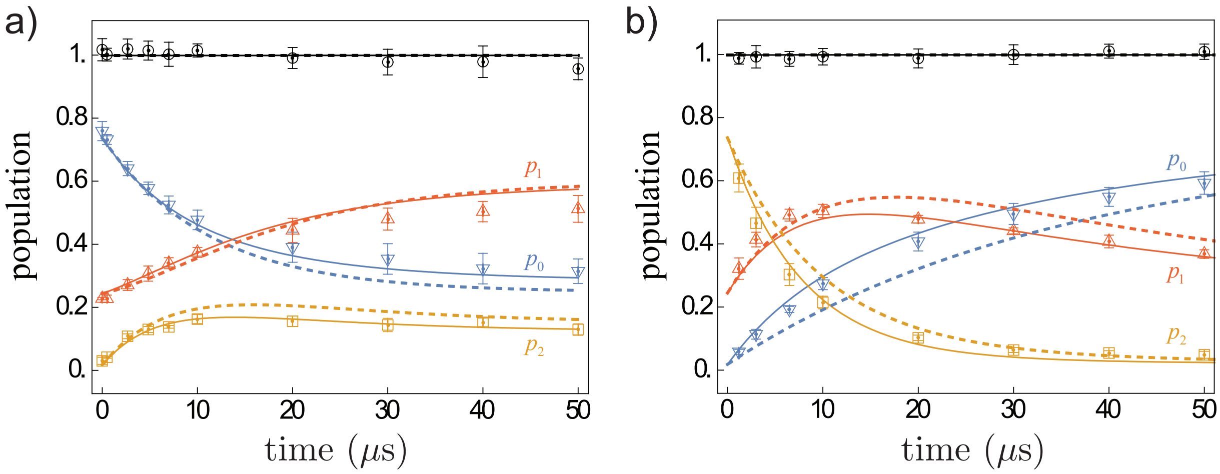


**Supplementary Figure 7: Comparing relaxation models.** (a) Time-domain evolution of populations during readout and (b) after a $|0\rangle$-$|2\rangle$ swap. The latter is better reproduced by the relaxation model with $x_{\mathrm{qp}} = 10^{-6}$ (plain lines) than with $x_{\mathrm{qp}} = 0$ (dashed lines), but the presence of quasiparticles has little influence on the time dynamics during fluorescence readout.

The decay times $T_{ij}^{\mathrm{rad,diel,qp}} = 1/(\Gamma_{ij}^{\mathrm{rad,diel,qp}} + \Gamma_{ji}^{\mathrm{rad,diel,qp}})$ computed from the above expressions for $Q_{\mathrm{diel}} = 5 \times 10^5$ and $x_{\mathrm{qp}} = 10^{-6}$ can be found in Supplementary Table 1. Importantly, they show that even though the parity selection rule is broken at $\phi_{\mathrm{ext}}/2\pi = 0.507$, all decays of even transitions are strongly suppressed for radiative and dielectric loss. Moreover, the presence of quasiparticles has a moderate effect on decoherence, and doesn't affect the qubit transition. Finally, note that the value of $Q_{\mathrm{diel}}$ gives a slightly lower qubit lifetime than measured experimentally (52 $\mu$s measured and 38 $\mu$s simulated).

As highlighted in the main text the existence of quasiparticle decay is not the limiting factor of the fluorescence dynamics. It is particularly clear when plotting the dynamics predicted by both relaxation models with $x_{\mathrm{qp}} = 10^{-6}$ and $x_{\mathrm{qp}} = 0$ (Supplementary Fig. 7). The presence of a non-zero quasiparticle population seems necessary to explain the decay from $|2\rangle$, but has very little effect on the time constant of $p_0(t)$ during readout.

| $T_{ij}^{\mathbf{rad}}$ ($\mu$s) | $\|1\rangle$ | $\|2\rangle$ | $\|3\rangle$ |
|---|---|---|---|
| $\|0\rangle$ | $8.1 \times 10^4$ | $3.0 \times 10^3$ | 0.091 |
| $\|1\rangle$ | | 142 | $1.0 \times 10^3$ |
| $\|2\rangle$ | | | 122 |
| $\|3\rangle$ | | | |

| $T_{ij}^{\mathbf{diel}}$ ($\mu$s) | $\|1\rangle$ | $\|2\rangle$ | $\|3\rangle$ |
|---|---|---|---|
| $\|0\rangle$ | 38 | $1.5 \times 10^4$ | 123 |
| $\|1\rangle$ | | 13 | $2.2 \times 10^5$ |
| $\|2\rangle$ | | | 3.6 |
| $\|3\rangle$ | | | |

| $T_{ij}^{\mathbf{qp}}$ ($\mu$s) | $\|1\rangle$ | $\|2\rangle$ | $\|3\rangle$ |
|---|---|---|---|
| $\|0\rangle$ | $1.2 \times 10^4$ | 33 | $1.1 \times 10^5$ |
| $\|1\rangle$ | | $1.7 \times 10^5$ | 36 |
| $\|2\rangle$ | | | $1.1 \times 10^5$ |
| $\|3\rangle$ | | | |

**Supplementary Table** 1: **Decay times** computed at $\phi_{\text{ext}}/2\pi = 0.507$ according to the model used to reproduce the data of Fig. 3 of the main text.

# Supplementary References


[1] N Bergeal et al. "Phase-preserving amplification near the quantum limit with a Josephson ring modulator". In: *Nature* 465.7294 (2010), p. 64. URL: http://www.nature.com/nature/journal/v465/n7294/abs/nature09035.html.

[2] G. Catelani et al. "Relaxation and frequency shifts induced by quasiparticles in superconducting qubits". In: *Physical Review B* 84.6 (Aug. 2011), p. 064517. ISSN: 1098-0121. DOI: 10.1103/PhysRevB.84.064517. arXiv: 1106.0829. URL: https://link.aps.org/doi/10.1103/PhysRevB.84.064517.

[3] A. A. Clerk et al. "Introduction to quantum noise, measurement, and amplification". In: *Reviews of Modern Physics* 82.2 (Apr. 2010), pp. 1155–1208. ISSN: 0034-6861. DOI: 10.1103/RevModPhys.82.1155. URL: http://link.aps.org/doi/10.1103/RevModPhys.82.1155%20https://link.aps.org/doi/10.1103/RevModPhys.82.1155.

[4] Nathanaël Cottet. "Energy and Information in Fluorescence with Superconducting Circuits". PhD thesis. 2019. URL: https://tel.archives-ouvertes.fr/tel-02002463.

[5] Jens Koch et al. "Charging Effects in the Inductively Shunted Josephson Junction". In: *Phys. Rev. Lett.* 103 (21 Nov. 2009), p. 217004. DOI: 10.1103/PhysRevLett.103.217004. URL: https://link.aps.org/doi/10.1103/PhysRevLett.103.217004.

[6] C Macklin et al. "A near-quantum-limited Josephson traveling-wave parametric amplifier". In: *Science* 350.6258 (Oct. 2015), pp. 307–310. ISSN: 0036-8075. DOI: 10.1126/science.aaa8525. URL: http://www.sciencemag.org/cgi/doi/10.1126/science.aaa8525.

[7] Easwar Magesan et al. "Efficient measurement of quantum gate error by interleaved randomized benchmarking". In: *Physical review letters* 109.8 (2012), p. 080505.

[8] K. Serniak et al. "Hot Nonequilibrium Quasiparticles in Transmon Qubits". In: *Physical Review Letters* 121.15 (Oct. 2018), p. 157701. ISSN: 0031-9007. DOI: 10.1103/PhysRevLett.121.157701. arXiv: 1803.00476. URL: http://arxiv.org/abs/1803.00476%20http://dx.doi.org/10.1103/PhysRevLett.121.157701%20https://link.aps.org/doi/10.1103/PhysRevLett.121.157701.

[9] Uri Vool et al. "Non-Poissonian quantum jumps of a fluxonium qubit due to quasiparticle excitations". In: *Physical Review Letters* 113.24 (2014), p. 247001.

[10] Chen Wang et al. "Measurement and control of quasiparticle dynamics in a superconducting qubit". In: *Nature Communications* 5 (2014), p. 5836.

[11] C Wang et al. "Surface participation and dielectric loss in superconducting qubits". In: *Applied Physics Letters* 107.16 (2015), p. 162601. ISSN: 1077-3118. DOI: 10.1063/1.4934486. URL: http://scitation.aip.org/content/aip/journal/apl/107/16/10.1063/1.4934486.
